# Does long-term care use within primary health care reduce hospital use among older people in Norway? A national five-year population-based observational study

**DOI:** 10.1186/1472-6963-11-287

**Published:** 2011-10-26

**Authors:** Trygve S Deraas, Gro R Berntsen, Toralf Hasvold, Olav H Førde

**Affiliations:** 1 Centre of Clinical Documentation and Evaluation, Northern Norway Regional Health Authority, Tromsø, Norway; 2 Institute of Community Medicine, University of Tromsø, Norway; 3 Norwegian Centre for Integrated Care and Telemedicine, University Hospital of North-Norway, Tromsø, Norway

## Abstract

**Background:**

Population ageing may threaten the sustainability of future health care systems. Strengthening primary health care, including long-term care, is one of several measures being taken to handle future health care needs and budgets. There is limited and inconsistent evidence on the effect of long-term care on hospital use. We explored the relationship between the total use of long-term care within public primary health care in Norway and the use of hospital beds when adjusting for various effect modifiers and confounders.

**Methods:**

This national population-based observational study consists of all Norwegians (59% women) older than 66 years (N = 605676) (13.2% of total population) in 2002-2006. The unit of analysis was defined by municipality, age and sex. The association between total number of recipients of long-term care per 1000 inhabitants (LTC-rate) and hospital days per 1000 inhabitants (HD-rate) was analysed in a linear regression model. Modifying and confounding effects of socioeconomic, demographic and geographic variables were included in the final model. We defined a difference in hospitalization rates of more than 1000 days per 1000 inhabitants as clinically important.

**Results:**

Thirty-one percent of women and eighteen percent of men were long-term care users. Men had higher HD-rates than women. The crude association between LTC-rate and HD-rate was weakly negative. We identified two effect modifiers (age and sex) and two strong confounders (travel time to hospital and mortality). Age and sex stratification and adjustments for confounders revealed a positive statistically significant but not clinically important relationship between LTC-rates and hospitalization for women aged 67-79 years and all men. For women 80 years and over there was a weak but negative relationship which was neither statistically significant nor clinically important.

**Conclusions:**

We found a weak positive adjusted association between LTC-rates and HD-rates. Opposite to common belief, we found that increased volume of LTC by itself did not reduce pressure on hospitals. There still is a need to study integrated care models for the elderly in the Norwegian setting and to explore further why municipalities far away from hospital achieve lower use of hospital beds.

## Background

Variations in hospital use can be explained by morbidity, health care system, socioeconomic and geographical factors [1-3]. Furthermore, variations in medical practice have an impact, especially on discretionary treatment of patients with conditions that have a limited evidence base related to diagnosis, treatment and follow-up. The prevalence of chronic, often multi-morbid patients increases with age and contributes to higher health care use among elderly people [4-6]. Hence, in the coming decades the expected ageing of the population and the reduced number of personnel available for both formal and informal care may potentially threaten the sustainability of the health care system [7,8].

Primary health care (PHC) can ideally take care of most elderly people with long-term needs and prevent illness or exacerbations. Consequently, several countries, including Norway, have planned to strengthen PHC, adopting the recommendations of the World Health Organization from 2008 [9-11]. Notwithstanding several inconsistencies, studies indicate that health care systems focusing on PHC have lower hospital use and score better with regard to access to health care, cost-effectiveness and mortality rates than systems focusing more on specialist health care [2,12-15].

However, most of the above-mentioned studies relate to a narrow definition of PHC, focusing specifically on the effect of physicians working within PHC. PHC has over the decades developed differently in various countries, reflecting their historical, cultural, and economic contexts as well as health care system [16]. In the Nordic countries most PHC is predominantly publicly funded, with varying levels of out-of-pocket co-payments, principally administered at the municipal level [17]. Municipal PHC is the frame in which all long-term care is carried out at and financed through, in contrast to many other countries where parts of long-term care are defined and financed as social care. Long-term care (LTC) is defined by the Organisation for Economic Co-operation and Development (OECD) as 'a range of services needed for persons who are dependent on help with basic activities of daily living' [18].

LTC in Norwegian municipalities includes formal home and community-based care including residential care homes and municipal nursing homes. The majority of home care is either nursing care in homes only or in combination with practical help [19]. Total home care users represent almost 75% of the total number of LTC-users aged 67 years and older [20]. Nursing homes and home care are under the same publicly funded financial system, carried out in the municipalities, where some services require co-payments depending on the care required. Nurse driven long-term care organizations are gate-keepers to municipal LTC, while general practitioners within a 'patient list' system ideally act as gatekeepers to specialist health care.

The OECD reports that insufficient long-term care services in Norwegian municipalities regularly require hospitals to take care of elderly people with no acute medical need [21]. Avoiding unnecessary or shortening hospital stays might reduce the use of this costly component of health care, and is also preferred by most patients with chronic disease [22]. However, the effects of LTC on hospitalization may go both ways. Long-term care may influence the utilization of acute and planned hospitalizations through several mechanisms: Reduced hospitalization by early detection of change in condition and needs, by enhancing psychosocial contact and support and by improved monitoring, care and treatment. Conversely, increasing the patient's need for medical treatment and functional support may also contribute to more hospitalization.

The proportion of formal home and community-based nursing services and municipal institutional care varies between Norwegian municipalities [20]. Such local differences are probably more influenced by organizational, economical, cultural and geographical factors, than by differences in morbidity and functional deficits. This illustrates the blurred boundaries between different LTC-categories from which identical patients might receive care from. In other words, different LTC-services in different municipalities are capable to give patient populations with similar medical/functional needs appropriate care and treatment [23,24]. As a consequence, it is indeed difficult to argue for any systematic differential effect of any LTC category of long-term care compared with another with respect to hospital bed use. We find that the best way of addressing LTC-use is to consider it as a municipal "LTC-package".

Furthermore, the relationship between hospital use and long-term care use is often studied among limited populations, geographical areas and diagnostic groups. The majority has studied the relationship between non-institutional LTC and hospital use. We have not found any studies on the relationship between municipal institutional care and hospital use. Consequently, as the evidence base for the assumption that LTC can prevent or shorten hospitalization is limited and conflicting, more research is needed [25,26].

We explored through a population-based observational study the following: How is use of LTC associated with hospital use? Is this association modified and/or confounded by sex, age, and/or other socioeconomic, demographic and geographic variables?

## Methods

This national population-based observational study consists of all Norwegians (59% women) older than 66 years (N = 605676) (13, 2% of total population) in 2002-2006. Each record in the database represents the population living within the same home-municipality, in the same sex and 5-year age-group. Thus, the unit of analysis is a municipal population group, and their concomitant average utilization rates of LTC and hospitalization over a 5 year period from 2002-2006. The outcome variable and main explanatory variable were aggregated from five year data (2002 to 2006) to one estimate pr variable pr unit of analysis for the time period. Data were linked from the following data sources: Norwegian Patient Registry (source 1), Statistics Norway (source 2) and all 25 hospital trusts (source 3). The study was approved by the Privacy Ombudsman for Research in Norway in accordance with the Personal Data Act and Health Registry Act (project number 17869).

### Outcome variable

Our measure of hospital use, HD-rate, was defined as the number of inpatient days in any Norwegian hospital per 1000 inhabitants for each unit of analysis (source 1).

### Explanatory variables

Our main explanatory variable, LTC-rate, was a composite variable consisting of number of recipients of municipal LTC (both at home and in institution) per 1000 inhabitants in each unit of analysis. It included the total number of recipients of LTC counted on a specific day each year in the age groups 67-74, 75-79, 80-84, 85-89 and 90-105 years (source 2). To obtain as many percentile groups as possible to visualize threshold effects, while avoiding unstable results due to small numbers in each group, we used six percentile groups. Percentile 1 represented the lowest 17% and percentile 6 the highest 17% of the LTC-rate within each age group.

We have further studied effect modifiers and confounding effects of the following variables known to affect hospital use:

• Municipal population size: Given both as number of inhabitants per unit of analysis and divided into six groups of municipality population size: 0-1999, 2000-4999, 5000-9999, 10 000-19 999, 20 000-49 999 and 50 000 or more inhabitants (in accordance with categories regularly used by Statistics Norway) (source 2).

• Hospital status: Defined as the municipalities hosting emergency hospital defined by services in at least internal medicine, surgery and radiology (source 3). As the hospital system changed somewhat over time we used status for 2004 for all years.

• Mortality: Age and sex specific all-cause mortality at the municipality level.

• Travel time: Travel time in minutes from municipality town hall to the closest emergency hospital (source 2).

• Municipality education level: Age and sex specific average proportion of the municipal population with primary school as highest education for the years 2002-2006 (source 2).

• Municipality relative poverty level: Average proportion of the population for the years 2005-2008 with a disposable household income below 60% of the median value [27] (source 2).

• Municipality unemployment level: Average proportion of the population 16-66 years that was unemployed for the years 2000-2009 (source 2).

### Statistical methods

Our model was developed in a stepwise forward manner, stratified by relevant effect modifiers and adjusted for relevant confounders. The outcome variable HD-rate has a Poisson distribution that approximates a normal distribution when the probability for the outcome is high (> 5%), thus allowing us to use a linear regression model in SPSS (Statistical Package for Social Sciences) v. 16 and SAS (Statistical Analysis System) v. 9.2. The relationship between the main explanatory variable (LTC-rate) and outcome variable (HD-rate), weighted by population size in each unit of analysis, was examined in a crude model, a model adjusted for age, a model adjusted for age and each of the variables in Table 1, and finally in a full model where all interacting and confounding variables were included.

**Table 1:** List of explanatory variables explored in the analyses

Explanatory variable	**Relationship to HD**-**rate?**	Effect modifier?	Confounder?	Included in final model?
**Sex**	HD-rates in Men > women	Yes	Not applicable	Stratifying variable
**Age**	Linear positive	Yes	Yes	Stratifying and adjustment variable
**Composite variable: Municipality population size and Hospital status**	HD-rates in hospital municipalities > Large municipalities without hospital > Small municipalities without hospital	No	Yes	Adjustment variable
**Mortality**	Linear positive	No	Yes	Adjustment variable
**Travel time to hospital**	Men 67-84 and women 67-79: no relationship. Men 85+ and Women 80+: Linear, negative	No	Yes	Adjustment variable
**Municipality education**	Men 67-84 and women 67-79: no relationship. Men85+ and Women 80+: Linear, negative	No	Yes	Adjustment variable
**Municipality relative poverty level**	Men 67-84 and women 67-79: no relationship. Men85+ and Women 80+: Linear, negative	No	No	Not included
**Municipality unemployment**	Linear positive	No	No	Not included

The variables in Table 1 were selected from a larger group of variables which theoretically or empirically have a relationship to either the explanatory and/or the outcome variable. They could therefore be both possible interacting and confounding variables, which had a statistically significant crude relationship to the outcome variable. We separately examined the possibility of interacting effects, and secondly confounding effects on the main relationship between LTC-rate and HD-rate for each of these variables.

Interactions: Given the large N in the dataset, almost all statistical tests of effect modification were highly statistically significant (p < 0.001). Using statistical criteria would yield an impossible number of interactions to deal with, most of which seemed to represent "white noise". To avoid an arbitrary selection of which variables had an interacting effect, we formalized criteria for clinically significant interaction: 1) Non-linear associations: the predicted least square means for HD-rates were plotted by LTC-rate percentile in line graphs, stratified by the possible interacting variable. The lines were transposed so that they all had one common point, and defined an interaction if trajectories differed by more than the clinically relevant change in outcome (1000 inpatient days per 1000 inhabitants) in two or more points. 2) Linear associations: Defined interaction if β-values of lines doubled or changed sign compared to the reference line.

Confounding was defined as a change in the predicted least square means of the model with and without the confounding factor of > 10%. To account for correlation within municipalities, we adjusted for municipality as a random effects variable. Finally we checked that the distributions of the standardized residuals for both the intermediate model (main variables, age and sex), and the final model were normally distributed thereby validating the choice of analysis method.

Descriptive background information is also presented to give the reader an understanding of how the main variables included in the analyses vary by the explanatory variable LTC and the stratification variables. Differences within groups for categorical variables are tested by Chi-square test and for continuous variables with ANOVA.

## Results

**Figure 1:**
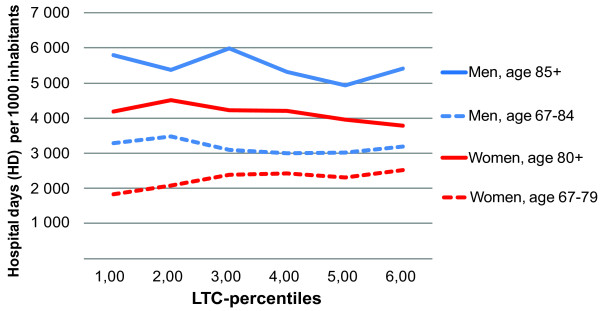
**Crude association percentiles of long-term care (LTC) rates and hospital day rates**. By age and sex group. Norwegian population. National average of years 2002-2006. 1^st ^percentile group represents the 17% lowest percentage in each 5-year age group.

Crude analysis showed a weak negative association between LTC-rate and HD-rate. The association was modified by sex. Effect modification plots made by sex, illustrated different association in younger age-groups compared to older age-groups. As relationships were linear, we plotted β-values for the five age groups for each sex. Guided by the change of sign for the β-values for the individual age groups, we stratified the data into the gender specific age-strata. For men: 67-84 years and 85 years and over. For women: 65-79 years and 80 years and over. The crude association stratified by these four age and sex groups is shown in Figure 1.

**Figure 2:**
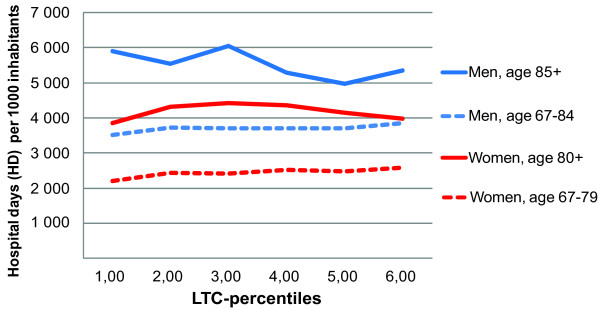
**Age-adjusted association long-term care (LTC) rates and hospital day rates**. By age and sex group. Norwegian population. National average of years 2002-2006. 1^st ^percentile group represents the 17% lowest percentage in each 5-year age group.

Adjustment for age: The analysis showed an interaction for both age and sex, which caused us to stratify analyses by these variables. Although we use age-specific LTC-percentiles, the age-distribution is quite different for male and female populations (see Table 2), which means that in the sex-stratified analysis there is no longer an even distribution of age. We therefore adjusted for age in the model, to correct for this skewness. Figure 2 illustrates the association between LTC-rates and HD-rates in the four sex and age groups when adjusted for age.

**Table 2:** Distribution of main exposure and outcome variables^a^.

	**Percentile - LTC group**								
				
		**1.00**	**2.00**	**3.00**	**4.00**	**5.00**	**6.00**	**All**	**P value**
	
**Population (*N*)**	Men, age 67-84	83212	73065	40454	11235	6079	8341	222386	< 0.000**
	Men, age 85-105	15050	8284	2218	820	777	915	28064	
	Women, age 65-79	3334	10311	28690	53965	62244	56606	215150	
	Women, age 80-105	861	9629	27184	33351	35234	33817	140076	
	
	**All**	**102457**	**101289**	**98546**	**99370**	**104333**	**99678**	**605676**	
	
**Rate LTC- users (users per 1000 inhabitants)**	Men, age 67-84	116	140	122	129	141	193	129	< 0.000*
	Men, age 85-105	488	548	688	776	768	951	553	< 0.000*
	Women, age 65-79	57	71	121	129	137	187	141	< 0.000*
	Women, age 80-105	691	657	506	551	573	655	581	< 0.000*
	
	**All**	**174**	**215**	**241**	**276**	**289**	**353**	**258**	< 0.000*
	
**Rate hospital days (days per 1000 inhabitants)**	Men, age 67-84	3289	3479	3090	2997	3013	3201	3289	< 0.000*
	Men, age 85-105	5807	5377	5995	5318	4932	5407	5643	< 0.000*
	Women, age 65-79	1838	2074	2386	2428	2301	2525	2385	< 0.000*
	Women, age 80-105	4188	4515	4223	4201	3949	3785	4063	< 0.000*
	
	**All**	**3619**	**3590**	**3263**	**3111**	**2919**	**3035**	**3256**	< 0.000*
	
**Travel time (minutes)**	Men, age 67-84	19.09	18.18	24.12	56.77	58.84	93.29	25.48	< 0.000*
	Men, age 85-105	17.79	30.00	36.02	41.91	66.84	69.91	26.60	< 0.000*
	Women, age 65-79	22.53	26.54	22.74	13.07	16.52	43.72	24.21	< 0.000*
	Women, age 80-105	42.22	18.84	17.80	16.25	20.19	44.09	24.60	< 0.000*
	
	**All**	**19.20**	**20.06**	**22.24**	**19.32**	**20.60**	**48.23**	**24.88**	< 0.000*
	
**Rate mortality (deaths per 1000 inhabitants)**	Men, age 67-84	48.00	50.22	40.11	38.29	38.17	43.02	46.35	< 0.000*
	Men, age 85-105	186.07	169.46	203.52	229.57	215.55	247.76	186.64	< 0.000*
	Women, age 65-79	13.14	13.83	21.44	21.19	20.18	23.33	21.02	< 0.000*
	Women, age 80-105	211.05	162.87	91.55	99.26	96.98	98.85	102.15	< 0.000*
	
	**All**	**68.52**	**66.98**	**52.54**	**51.05**	**48.62**	**52.66**	**56.76**	< 0.000*

Norwegian population. Average for 2002-2006

^a ^By age-specific percentiles of long-term care (LTC-) rates in men and women aged 67 years and older.

1st percentile group represents the 17% lowest percent in each defined age and sex strata.

*P values for within-group differences. One-way ANOVA.

**P value for within-table differences. Chi-squared test.

***Absolute rates of LTC-use in each age-sex stratum by LTC-percentile and age-sex groups

The national distribution of population, HD-rates and LTC-rates by these age and sex groups is depicted in Table 2. The effect modification and/or confounding effects of other adjustment variables are listed in Table 1.

We identified two important confounders:

1) Travel time: Smaller municipalities far away from their local hospitals, still had the same positive relationship between LTC-rates and HD-rates. However, including this variable changed the main association from weakly negative to weakly positive indicating a confounding of the main association.

2) Mortality: For men 85+, mortality is higher in the high LTC-groups, whereas for women 80+, mortality is higher in the lower LTC-groups. Adjusting for mortality in the two older age groups maintained or increased their negative relationships between LTC- and HD-rates.

**Figure 3:**
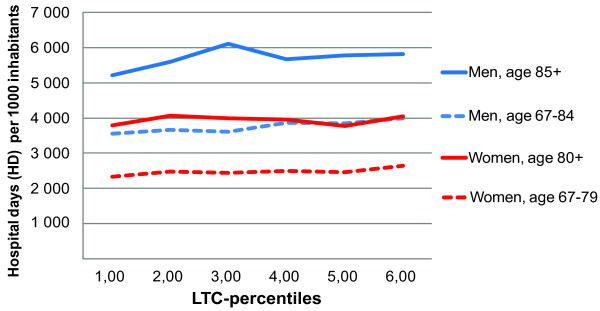
**Fully adjusted model^a^: Association between long-term care (LTC) rates and hospital day rates**. By age and sex. Norwegian population. National average across years 2002-2006. 1^st ^percentile group represents the 17% lowest percentage in each 5-year age group. ^a^Adjusted for age, mortality, travel time to hospital, education, and composite variable of "municipality population size" and "hospital status", with municipality as random factor.

All confounders had a linear relationship with the outcome variable and were included as covariates. The relationship between LTC-rates and HD-rates by population stratum and model variations as described by β-values is given in Table 3. It is interesting to note that in the crude analyses, the relationship is negative in three of the four strata. This relationship remains negative in the two elder age groups when adjusted for age and mortality. Thus, in the model adjusted only for age and mortality, increasing LTC is associated with an almost clinically significant reduction of HD-rates in the two oldest age-groups. Using β-values (Table 3), we calculated a difference of -612 and -665 HD per 1000 inhabitants between the lowest and highest LTC-percentiles in men 85+ and women 80+ respectively from the age-mortality adjusted model. However, adjusting for travel time from hospital changes this picture around. In the full model where all significant variables are included the β-values for the three positive lines were significantly different from zero (p < 0,001), whereas the negative line was not (p < 0.4) (Table 3 and Figure 3). Therefore, it seems that the higher LTC-use and lower hospital use in municipalities far away from hospital is the stronger confounder. The gap in hospitalization rates between the lowest and highest long-term care percentiles was less than 1000 days per 1000 inhabitants in all age and sex groups. Analyses without mortality in the full model did not change the above relationships.

**Table 3:** β-values^a ^for assumed linear relationship between percentiles of long-term care (LTC) rates and hospital day rates^b^

Sex and age group	Crude	Adjusted for age	Adjusted for age, travel time	Adjusted for age, mortality	Fully adjusted model
	(95% CI)	(95% CI)	(95% CI)	(95% CI)	(95% CI)
**Men, age 67-84**	-64.02*	64.58***	92.00***	44.91**	76.99***
	(-110.3 - -17.7)	(34.8 - 94.4)	(59.2 - 124.7)	(14.7-75.2)	(44.3-109.7)
**Men, age 85+**	-114.93*	-139.53**	**9.07**	-122.48*	142.36***
	(-214.1 - -15.7)	(-238.9 - -40.1)	(-95.5 - 113.6)	(-221.5 - -23.l3)	(58.3 - 226.5)
**Women, age 67-79**	68.90***	47.10***	56.66***	32.45***	52.47***
	(34.2 - 103.8)	(23.2 - 71.0)	(32.1 - 81.2)	(8.3-56.5)	(25.7 - 79.2)
**Women, age 80+**	-162.30***	-112.37***	**-45.64**	-133.10***	**-16.14**
	(-211.6- -112,9)	(-160.1 - -64.6)	(-93.1 - 1.8)	(-179.9- -86.3)	(-54.0 - 21.7)

^a ^Adjusted for age, mortality, travel time to hospital, education, composite variable of "municipality population size" and "hospital status", with municipality as random factor.

^b ^By age-specific percentiles of long-term care (LTC-) rates in men and women aged 67 years and older.

P values: *** for p < 0,001, ** for p < 0,01 and * for p < 0, 05

## Discussion

Our principal finding was an overall weak positive statistically significant, but still not clinically important relationship between use of long-term care and hospital use for women aged < 80 years and men in all age groups. For women 80 years and older the weak negative association was neither statistically significant nor clinically important. The influence of travel time to hospital had more impact than mortality as the main confounders in the final analyses.

### Strengths

Our analysis was strengthened by the large, robust and comprehensive national dataset broken down into relatively high numbers of units of analysis that offer a suitable base to study associations between explanatory factors and health care use. While we had age- and sex-specific strata we were able to examine effect modification of age and sex.

Norway's 430 municipalities (2008) are well defined administrative units most frequently used in public statistics and are also responsible for the provision of PHC including long-term care. We consider the outcome measures to be of good quality as they are also used for financial purposes and thus checked and re-checked by hospitals, the Norwegian Patient Registry and finally by various researchers without revealing any systematic misclassification. The degree of misclassification and annual municipal variation were minimized by using 5-year aggregated measures.

Our measure of hospital use, the HD-rate, can be broken down into both the rate of admissions and the average number of days in hospital per stay, which both contribute to the total use of resources in inpatient secondary care. Data from Statistics Norway are derived from national public registries of all citizens of Norway. The data on long-term care in the municipalities include almost all recipients, as the private sector is minimal. The data has been through an internal quality check mainly based on comparison with previous year's data and internal consistency. The rates of home care recipients and institutional residents were highly correlated (r = 0.71), which strengthens our argument for a composite measure of LTC recipients. Inclusion of geographical, socioeconomic and demographic variables made it possible to adjust for characteristics of municipality and inhabitant groups. As the Norwegian health care system has given PHC a high priority over the last decades, our findings are relevant to other countries that plan to strengthen PHC and to integrate social care with health care.

### Limitations

The criterion for clinical significance was a post hoc discretionary decision made by the authors. We needed a limit which could make clinical sense, and we found that change of a length of hospital stay by a day per person (1000 hospital days per 1000 inhabitants), is a unit which is easily grasped by patients, health managers and clinicians alike. Using statistical significance alone, almost all the variables in Table 1 would be identified as interacting variables, causing an impossible number and hardly meaningful number of stratifications. By choosing a relatively strict definition for clinical significance, we have here taken into account only the strongest interactions apparent in the data.

In our study, aggregated data represent the average effects of individuals in each unit of analysis. The optimal design using individual data were not feasible due to the strict privacy legislation in Norway.

Data from Norwegian Patient Registry are collected for financial purposes, and therefore may be biased in the direction of increased earnings for hospitals. The LTC-data are collected for administrative purposes, but are not linked to financial incentives. A possible bias could be higher reporting of services than what is actually produced to justify supplier capacity, which is a phenomenon called gaming.

However, there is no reason to believe that if these variables are biased this way, which should be differential or dependent on the other variable in the association. Thus, such an undifferentiated bias would tend to move results towards the null hypothesis - no relationship, which means that our results may be an underestimate of the real association.

Hospital days in private hospitals are not included in our data. According to official statistics they represent a mere of 0.8% of the total hospital days. Hence the hospital days data represent a minor source of error [28]. Data on private outpatient clinics that in some specialities which especially in urban areas provide significant parts of the total outpatient volume was not available for this time period [29]. Ambulatory day-based hospital services were under implementation in the health service in this period and thus varied highly between hospitals. Therefore, since in-patient stays is the most expensive component of secondary care, and in relation to LTC the most relevant, we judged hospital days to be the most appropriate measure for utilization of secondary care services.

Due to lack of data of morbidity, all-cause mortality in our ten age and sex groups was used as a proxy for morbidity. It is thus both a marker for general conditions related to mortality and LTC need in the local community. It could be argued that morbidity, or as in our case mortality, is part of the line of causation between LTC-rates and HD-rates, and including it in the model would then be an over-adjustment [30]. However, analyses without mortality in the full model did not change the relationships in the fully adjusted model.

### Previous research

To the best of our knowledge only a few publications analyse the associations that we have studied. An American publication at a national level found a positive association at both state and hospital referral region level between rates of use of hospital inpatient facilities and use of long-term care when adjusted for age, sex, race and chronic illness [31]. A Swedish study showed that people receiving municipal long-term care had higher hospital use than those not receiving such care [26]. The study consisted of 4907 people aged 65-104 years from four municipalities in the south of Sweden. The study was undertaken in a health system almost identical to the Norwegian one. But, although the sample was on individuals, the sample size was small compared with ours, which is probably why they were unable to adjust for possible effect modifiers or confounders. A Norwegian study using a linear regression model showed reduced length of stay with increased long-term care. The study used three-years data, and the municipalities' expenditures on long-term care as a measure of long-term care capacity and adjusted case-mix by using a "Diagnosis Related Group (DRG) index", which may represent an over-adjustment of case-mix [32]. Interestingly, we also found a crude negative association between long-term care and hospital use. However, the association changed direction in the fully adjusted model which is more in line with the two first mentioned studies.

Studies of the effect of specific subdivisions of LTC on hospital use demonstrate variable results. A Canadian study found that home care recipients had five-fold higher hospital inpatient days than among well elderly people and long-term care residents, perhaps illustrating a higher morbidity and vulnerability [33]. The study used individual data on a total of 41803 people aged 65 and older, but did not control for any effect modifiers or confounders, neither for age nor sex differences. An American study found that a higher number of home care visits were positively associated with hospitalization independent of rurality, whereas another small American study showed a benefit of home care [34,35]. A meta-analysis from 1997 showed that home care reduced, although only moderately, emergency hospital days use among elderly people [25]. Home care was also associated with a lower risk of hospitalization. However, the effect size was relative moderate in most studies.

### Interpretation of the results

Our findings could be interpreted as long-term care use has no relation to hospitalization. However, as this is an observational study, where all municipalities are required to offer LTC, there is no control population without LTC, hence we do not know how the HD-rate would be in areas completely without or with much lower LTC-use.

LTC-services are by definition provided to people with a functional loss, who also may have significant morbidity and hospitalization needs. Depending on the threshold for LTC the LTC-rates could be markers for different health care needs. In this material, we only had mortality as a marker for morbidity in the LTC-groups making it difficult to compare groups of the same morbidity with high and low LTC with one another. We cannot be certain that confounding by morbidity is completely removed from the analyses, and that LTC remains mainly a marker of the need for functional and/or medical assistance.

If the threshold for LTC-inclusion was lower, also smaller health care needs could lead to LTC-inclusion and we might expect a higher preventive effect of LTC on hospitalization, because these needs are less complex and easier to meet in a LTC-setting. If this was true, LTC would be more than a marker for co-morbidity and hospitalization needs. We would then expect the crude association to be negative, which is what we did find. However, for this to be a conclusive interpretation, it would have to remain negative also after adjustments. We found that adjustment for travel time to hospital turned the picture around. It was the high LTC-rates and low HD-rates in rural municipalities that caused the unadjusted association to be negative. After adjustment for this factor, the association turned flat to weakly positive.

If the threshold for LTC-inclusion is in-between two extremes, the gradient would change to one that is less positive or flat. It is however impossible without a control group, to determine the strength of this theoretical effect. Therefore the relatively weak positive association found in this study does not preclude some preventive effect of LTC on hospital use.

Even the highest LTC-rate groups with highest HD-rates in the smaller municipalities situated far away from hospital, had lower HD-rates than the lowest LTC- and HD-rates in hospital municipalities given the same morbidity. We speculate that municipalities with a long distance to secondary care may have organized and integrated their municipal care different from hospital municipalities.

There is a need to extend the knowledgebase before making political decisions on strengthening long-term care in an attempt to reduce hospitalization, also within a health care system that has had a significant and stable primary health care focus for decades.

Recent developments of integrated care models including long-term care show better results regarding the need of hospitalization when the entire team of health care professionals collaborate to meet the patient's needs [36]. Consequently we recommend studies in areas selected on basis of high or low HD-rates or LTC-rates. The studies should use individual patient data including morbidity and also focus on total care resource use including "business case" evaluations [37,38]. As frail elderly people often benefit from and also prefer to receive more seamless care closer to home than they do currently, even an equal business case can be seen as progress for these patients [22,25].

Further research should also focus on transitions between levels of care and variations of delivery models in the local setting [39]. The latter should focus a multidimensional evaluation of the elderly needs, continuity and coordination of care as well as teambuilding between different professionals and informal caregivers [40]. As models from other health care systems are not necessarily transferable to the Scandinavian model of primary health care, the effect of such models for improvement of elderly care must be studied and proven to be cost-beneficial in the Norwegian context [41].

## Conclusions

After adjustment for confounding factors a weak negative crude association between rates of long-term care recipients and hospital days turned to a weak positive adjusted association. The influence of travel time to hospital had more impact than mortality as a confounder in the final analyses. Although our findings have several possible interpretations, we warn against the strong belief in several countries that an increased volume of LTC by itself will reduce pressure on hospitals. There still is a need to study the effect of integrated and chronic care models for the elderly in the Norwegian setting and to explore further why municipalities far away from hospital achieve lower use of hospital beds.

## Competing interests

The authors declare that they have no competing interests.

## Authors' contributions

TSD and GB initiated, planned the study and collected the data. GB and TSD carried out the data analyses. TSD has been the primary author of the manuscript, while all other authors contributed to the writing of the manuscript and read and approved the final manuscript.

## Pre-publication history

The pre-publication history for this paper can be accessed here:

http://www.biomedcentral.com/1472-6963/11/287/prepub
